# Multimorbidity Patterns and Memory Trajectories in Older Adults: Evidence From the English Longitudinal Study of Aging

**DOI:** 10.1093/gerona/glab009

**Published:** 2021-01-15

**Authors:** Rebecca Bendayan, Yajing Zhu, Alex D Federman, Richard J B Dobson

**Affiliations:** 1 Department of Biostatistics and Health Informatics, Institute of Psychiatry, Psychology and Neuroscience, King’s College London, UK; 2 NIHR Biomedical Research Centre at South London and Maudsley NHS Foundation Trust and King’s College London, UK; 3 Personalized Healthcare, Product Development, F.Hoffmann - La Roche Ltd, Cambridge, UK; 4 MRC Biostatistics Unit, University of Cambridge, UK; 5 Division of General Internal Medicine, Department of Medicine, Icahn School of Medicine at Mount Sinai, New York, New York, USA

**Keywords:** Cognitive decline, Longitudinal, Multiple health conditions

## Abstract

**Background:**

We aimed to examine the multimorbidity patterns within a representative sample of UK older adults and their association with concurrent and subsequent memory.

**Methods:**

Our sample consisted of 11 449 respondents (mean age at baseline was 65.02) from the English Longitudinal Study of Aging (ELSA). We used 14 health conditions and immediate and delayed recall scores (IMRC and DLRC) over 7 waves (14 years of follow-up). Latent class analyses were performed to identify the multimorbidity patterns and linear mixed models were estimated to explore their association with their memory trajectories. Models were adjusted by sociodemographics, body mass index (BMI), and health behaviors.

**Results:**

Results showed 8 classes: Class 1: Heart Disease/Stroke (26%), Class 2: Asthma/Lung Disease (16%), Class 3: Arthritis/Hypertension (13%), Class 4: Depression/Arthritis (12%), Class 5: Hypertension/Cataracts/Diabetes (10%), Class 6: Psychiatric Problems/Depression (10%), Class 7: Cancer (7%), and Class 8: Arthritis/Cataracts (6%). At baseline, Class 4 was found to have lower IMRC and DLRC scores and Class 5 in DLRC, compared to the no multimorbidity group (*n* = 6380, 55.72% of total cohort). For both tasks, in unadjusted models, we found an accelerated decline in Classes 1, 3, and 8; and, for DLRC, also in Classes 2 and 5. However, it was fully attenuated after adjustments.

**Conclusions:**

These findings suggest that individuals with certain combinations of health conditions are more likely to have lower levels of memory compared to those with no multimorbidity and their memory scores tend to differ between combinations. Sociodemographics and health behaviors have a key role to understand who is more likely to be at risk of an accelerated decline.

Multimorbidity, defined as the coexistence of 2 or more health conditions, is the norm in primary care (1,2) and its prevalence is estimated to reach 67.8% of UK population over 65 years old by 2035 (3), from which nearly half will include cognitive impairment, depression, or dementia (3). Most research to date exploring the association between physical health conditions and cognitive decline focuses on single or specific chronic physical diseases, particularly cardiovascular diseases (eg, 4). Fewer studies have examined the impact of multimorbidity in cognitive performance in older adults (5–10). Cross-sectional studies have found that having 2 or more co-existent conditions (5) or 4 or more conditions (6) was associated with greater risk of mild cognitive impairment (MCI). For example, Poblador-Plou et al. (8) went beyond cutoffs and explored patterns of multimorbidity in a sample of Spanish individuals over 65 years old. They found that diabetes and hypertension were the most frequent chronic health conditions in individuals with dementia. However, when they identified patterns of multimorbidity, the only pattern associated with dementia did not include diabetes or hypertension but Parkinson’s disease, congestive heart failure, cerebrovascular disease, anemia, cardiac arrhythmia, chronic skin ulcers, osteoporosis, thyroid disease, retinal disorders, prostatic hypertrophy, insomnia, anxiety, and neurosis.

Longitudinal research exploring the association between multimorbidity and cognitive decline is limited and results are still inconsistent. Some studies found that having 2 or more health conditions was associated with a higher risk of MCI or dementia after 5 years (9), faster cognitive decline over 5 years but only for individuals with dementia (7). Other longitudinal studies explored multimorbidity as a predictor of cognitive decline, but these were limited as they only considering cardiovascular comorbidities (4) or used weighted indexes (10). Although these studies found that multimorbidity was a predictor of accelerated decline in cognition, results varied depending on the specific cognitive domain that was examined. For example, Wei et al. (10) found that each point increase in their multimorbidity index was associated with faster decline in immediate recall tasks but not delayed recall tasks. All these studies understood multimorbidity in terms of quantity or focused on prespecified patterns and only 2, to our knowledge, explored the differential association of each specific patterns of multimorbidity with cognition. Aarts et al. (11) examined the association between 96 chronic diseases, grouped into 23 disease clusters, and cognition over 6 and 12 years of follow-up using data from adults ranging 24–81 years old. These authors defined multimorbidity as the combination of identified disease clusters and they found that some specific disease clusters had a greater association with cognition than the combinations of disease clusters (ie, multimorbidity). When these authors looked at cluster combinations, they found that only the combination of malignancies and movement disorders was significantly related to cognitive decline. Bratzke et al. (12) explored the association between chronic illnesses and MCI finding that those individuals that had sleep disturbances, apnea, and cholesterol were the only ones that showed a significant decline in memory, defined as amnesic MCI, over 8 years follow-up. However, decline was not evident for individuals assigned to the depression class (cholesterol, depression, and arthritis) or the cardiovascular class (cholesterol, hypertension, and arthritis). Within this context, there is need for further research to identify whether individuals with specific health profiles are at greater risk of accelerated cognitive decline. Specifically, we examine the different patterns of multimorbidity within a representative sample of UK older adults and examine their association with concurrent and subsequent memory using immediate and delayed recall tasks.

## Method

### Setting and Sample

The English Longitudinal Study of Aging (ELSA) is an ongoing, nationally representative population-based longitudinal cohort of UK older adults. A total of 11 449 respondents (of which 9424 were complete cases and 7468 were alive after Wave 7) were included in the study, contributing to 49 757 person-wave observations. Individuals with intermittent missing data on cognition were not excluded from the study, unless no information was available across 7 waves (*N* = 183 and 186 for IMRC and DLRC, respectively). In this sample, each subject had an average of 4 waves of cognition data and 69% of subjects have at least 3 waves of data. This study aimed to capture normative cognitive decline and therefore individuals with self-reports of Alzheimer’s or dementia at baseline were excluded (*n* = 69).

### Health Conditions

Physician-diagnosed diseases were assessed at wave 1. ELSA study included those health conditions that were found to be the main conditions experienced by people in middle and older age and shown to be among the major sources of long-standing illness for people aged 65 years and older in other national surveys (details can be found in https://www.elsa-project.ac.uk/data-and-documentation). Participants were asked, “Has a doctor ever told you that you have…?” Fourteen assessed conditions were included: hypertension, diabetes, cancer, lung disease, heart problem, stroke, psychiatric problems, arthritis, asthma, high cholesterol, cataracts, Parkinson’s, hip fracture, and depression. Depression was defined using data from the 8-item version of the Center for Epidemiologic Studies Depression scale (CES-D; 13,14), which is a questionnaire developed as a depressive symptoms screening scale for epidemiological investigation. A cutoff of 4 or more symptoms on the 8-item scale to determine case-level depression which has been extensively used in previous aging research (eg, 15,16). All these conditions were coded as binary responses. These conditions are standardly collected in aging population studies which facilitates cross-cohort comparisons and replicability.

### Cognitive Performance

Cognitive performance was assessed via tests of immediate and delayed recall of 10 common nouns. Respondents were asked to recall as many words as possible immediately after the list was read and then again after around a 5-minute delay during which they completed other survey questions. Immediate and delayed recall tests have been shown to have good construct validity and consistency (17). Total scores are the number of words recalled in each test and range between 0 and 10, with higher scores indicative of better memory.

### Covariates

Sociodemographic variables such as age, sex, education, marital status, and wealth at baseline were included. Education was coded in a 3-tier harmonized scale (less than lower secondary education, upper secondary and vocational training, and tertiary education). Marital status was a binary indicator for married/in partnership and otherwise. Wealth was recorded using total family wealth (including net value of primary residence, business, and non-housing financial wealth) and divided into 5 quintiles. Health behaviors and BMI are known risk factors for cognitive decline (18). Health behaviors included smoking (ever smoked nor not) and physical activity (vigorous, moderate, light, and never). BMI values were categorized as obese or not using the standard cutoff of 30 (WHO definition).

### Statistical Analysis

In order to identify patterns of multimorbidity from the 14 health conditions recorded in Wave 1, we used latent class analysis (LCA). Under the missing at random assumption, individuals with missing values on these conditions were not excluded from the study (19). Following Nylund (20), the best-fit models were selected by considering a set of goodness of fit indexes which included log-likelihood, Bayesian information criterion (BIC), sample-sized adjusted BIC (SABIC), deviance, and relative entropy (range between 0 and 1 with higher values indicating good classification quality). Although class labels are usually defined by the set of conditions with the highest conditional probabilities, we find that in a multimorbidity settings, labels are easily dominated by prevalent conditions such that rarer co-occurring conditions were masked. To bring out the unique feature of each cluster, we estimated within-class prevalence and within-MM population prevalence for each cluster. To facilitate interpretation, each cluster is labeled by those conditions where the within-class prevalence is higher than the population prevalence. Each individual is assigned to a unique cluster where the probability of class membership is the highest (ie, modal class approach (20). Each cluster has a unique profile of the full set of comorbidities. For example, individuals in class A and B could both have X and Y conditions, but the majority of individuals assigned to class A have condition X while the majority of individuals assigned to class B have condition Y. This analytical approach which has been previously used in multimorbidity research (21) allows us to understand multimorbidity from an individualized perspective instead of from a disease-centric perspective. This is particularly relevant for personalized medicine.

In order to examine the association between multimorbidity clusters and subsequent changes in cognitive performance, linear mixed models were performed. To examine the trajectories of immediate and delayed recall over time linear mixed models with random coefficients (22) were estimated. Model 1 only included the multimorbidity classes (unadjusted model). Model 2 was adjusted by sociodemographics (age, gender, marital status, family wealth, and education level) and Model 3 was additionally adjusted by BMI and heath behaviors (smoking and physical exercise). Across all models, baseline age was centered at mean such that intercept reflected the initial cognition level of cohort member with a population-average age. The best-fit model was selected by considering a set of mode-fit statistics (AIC, BIC, Root-Mean-Square Error of Approximation [RMSEA], Comparative Fit Index [CFI], Standardized Root-Mean-Square Residual [SRMR], Non-normed Fit Index [NNFI], and likelihood ratio tests) (23).

Where covariates were missing (smoking [*n* = 149, 1%], physical activity [*n* = 153, 1%], marital status [*n* = 2, <1%], total wealth [*n* = 200, 2%], education [*n* = 999, 9%], BMI [*n* = 859, 8%]), these were imputed using multiple imputation by chained equations (24). Analyses were performed across 50 imputed datasets and combined using Rubin’s rules (25).

To evaluate the robustness of our results, a set of sensitivity analyses were performed. First, we compared results for individuals stratified in 2 age groups (50–69 and 70+) and compared results of complete-case analysis and imputed datasets. Second, we assessed the survival effect by comparing results from samples with and without cohort members who died before wave 7.

## Results

### Exploring Patterns of Multimorbidity: Latent Class Analysis

Preliminary exploratory analyses were performed to describe the disease profile for the non-multimorbid cluster (ie, those individuals that had none or a single condition). This group consisted of 6380 individuals with zero or only one condition (46% and 54%, respectively), where the 5 most prevalent were high blood pressure (32%), arthritis (23%), heart disease (9%), depression (8%), and asthma (8%).

**Figure 1.**
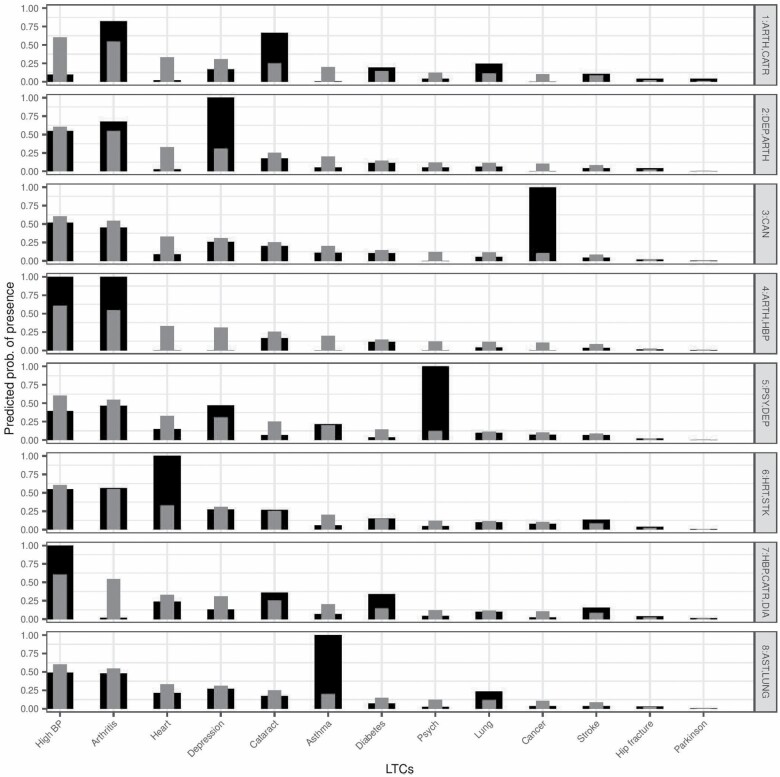
Probabilistic disease profile for each class. Class 1: HRT, STK (*n* = 1318); Class 2: AST, LUNG (*n* = 794); Class 3: ARTH, HBP (*n* = 635); Class 4: DEP, ARTH (*n* = 598); Class 5: HBP, CATR, DIA (*n* = 531); Class 6: PSY, DEP (*n* = 527); Class 7: CAN (*n* = 356) and Class 8: ARTH, CATR (*n* = 310). Black = estimated within-class prevalence. Gray = within-MM population prevalence. Clusters are labeled by the conditions of which the estimated within-class prevalence is higher than within-MM population prevalence.

Results of the LCA considering the individuals with multimorbidity (ie, 2 or more conditions) showed that the 8 classes model showed the lowest BIC and clear separation of classes (high entropy of 0.85). Fit indexes are shown in Supplementary Figure 1. We present clusters ordered by descending prevalence and labeled by those health conditions where the within-class prevalence was higher than the population prevalence. Class 1: Heart Disease/Stroke (26%), Class 2: Asthma/Lung Disease (16%), Class 3: Arthritis/Hypertension (13%), Class 4: Depression/Arthritis (12%), Class 5: Hypertension/Cataracts/Diabetes (10%), Class 6: Psychiatric Problems/Depression (10%), Class 7: Cancer (7%), and Class 8: Arthritis/Cataracts (6%). These labels represent those conditions that were the most prevalent in each group of individuals which allowed us to have described how these conditions tend to cluster in individuals. However, individuals in each class may have had also other conditions which were not highly prevalent in their group. For example, the majority individuals in Class 1 had heart disease and stroke and some individuals might have had also other conditions which were not highly prevalent in Class 1 such as arthritis. This approach allowed us to consider between individual variability which is essential for personalized medicine and more likely to represent what can be found in clinical settings. Please see Figure 1 for complete probabilistic disease profile.

Descriptive statistics for total sample and each cluster are given in Table 1. Chi-squared tests (*p* < .001) showed significant differences between clusters for each covariate. Although individuals in most classes were significantly older than the reference group (Supplementary Table 1), individuals in Class 8: Arthritis/Cataracts were on average the oldest followed by those from Class 1: Heart Disease/Stroke, while individuals from Class 6: Psychiatric Problems/Depression were the youngest (even compared with the reference group). Sex differences were also found across classes. Class 5: Hypertension/Cataracts/Diabetes had greater prevalence of men compared to the group without multimorbidity and Class 4: Depression/Arthritis had greater prevalence of women. Class 4: Depression/Arthritis and Class 8: Arthritis/Cataracts were more likely to have the lowest levels of education while the reference category, Class 6: Psychiatric Problems/Depression and Class 7: Cancer showed the higher levels of education. Class 4: Depression/Arthritis was the Class with higher economic disadvantage while the reference category, Class 5: Hypertension/Cataracts/Diabetes and Class 7: Cancer where those with less economic disadvantage. With regards to health-related variables, we found that individuals in multimorbidity clusters showed higher levels of light of physical activity and lower levels of vigorous physical activity when compared to the reference group. Moreover, we found that individuals in Class 3: Arthritis/Hypertension (13%), Class 4: Depression/Arthritis were the most likely to be obese.

**Table 1.** Descriptive Statistics for Total Sample (*N* = 11 449) and Classes

	Total	Class 0: No MM	Class 1: HRT, STK	Class 2: AST, LUNG	Class 3: ARTH, HBP	Class 4: DEP, ARTH	Class: 5: HBP, CATR, DIA	Class 6: PSY, DEP	Class 7: CAN	Class 8: ARTH, CATR
*N* (%)	11 449	6380 (55.72)	1318 (26)	794 (16)	635 (13)	598 (12)	531 (10)	527 (10)	356 (7)	310 (6)
Age (mean and *SD*)	65.02 (10.24)	62.48 (9.34)	71.27 (10.14)	65.56 (9.72)	68.06 (9.45)	67.10 (10.90)	69.54 (9.50)	60.92 (8.81)	68.95 (9.95)	73.69 (10.05)
Gender (male)	5193 (45%)	3083 (48%)	653 (50%)	297 (37%)	222 (35%)	202 (34%)	290 (55%)	182 (35%)	132 (37%)	132 (43%)
Smoking (ever smoked)	7266 (64%)	3862 (61%)	906 (70%)	536 (68%)	389 (62%)	402 (67%)	363 (69%)	359 (69%)	244 (69%)	205 (68%)
Physical activity										
Never	959 (8%)	283 (4%)	222 (17%)	101 (13%)	52 (8%)	104 (17%)	57 (11%)	48 (9%)	38 (11%)	54 (18%)
Light	1203 (11%)	394 (6%)	237 (18%)	124 (16%)	90 (14%)	112 (19%)	65 (12%)	69 (13%)	57 (16%)	55 (18%)
Moderate	4970 (44%)	2740 (44%)	551 (43%)	335 (43%)	302 (48%)	262 (44%)	260 (50%)	233 (45%)	154 (44%)	133 (44%)
Vigorous	4164 (37%)	2879 (46%)	222 (17%)	224 (29%)	183 (29%)	119 (20%)	141 (27%)	170 (33%)	104 (29%)	61 (20%)
Marital status (Single)	3511 (31%)	1561 (24%)	513 (39%)	288 (36%)	209 (33%)	291 (49%)	172 (32%)	205 (39%)	134 (38%)	138 (45%)
Wealth										
Quintile 1 (lowest)	2264 (20%%	906 (14%)	386 (30%)	214 (27%)	126 (20%)	203 (34%)	113 (22%)	166 (32%)	66 (19%)	84 (27%)
Quintile 2	2244 (20%)	1123 (18%)	306 (24%)	175 (22%)	143 (23%)	149 (25%)	117 (22%)	94 (18%)	71 (20%)	66 (21%)
Quintile 3	2256 (20%)	1301 (21%)	239 (18%)	149 (19%)	138 (22%)	106 (18%)	103 (20%)	92 (18%)	74 (21%)	54 (18%)
Quintile 4	2226 (20%)	1376 (22%)	194 (15%)	134 (17%)	118 (19%)	81 (14%)	98 (19%)	83 (16%)	83 (23%)	59 (19%)
Quintile 5 (highest)	2259 (20%)	1544 (25%)	174 (13%)	115 (15%)	98 (16%)	51 (9%)	92 (18%)	81 (16%)	60 (17%)	44 (14%)
Education										
Less than secondary	4868 (47%)	2283 (39%)	706 (60%)	406 (55%)	305 (53%)	371 (67%)	259 (53%)	205 (44%)	159 (50%)	174 (62%)
Upper Secondary	4303 (41%)	2679 (46%)	401 (34%)	252 (34%)	228 (40%)	161 (29%)	185 (38%)	198 (42%)	116 (37%)	83 (30%)
Tertiary	1279 (12%)	880 (15%)	79 (7%)	80 (11%)	41 (7%)	24 (4%)	41 (8%)	68 (14%)	42 (13%)	24 (9%)
Body mass index										
Normal	7779 (73%)	4685 (78%)	807 (69%)	510 (69%)	342 (59%)	345 (64%)	326 (67%)	341 (69%)	233 (70%)	190 (73%)
Obese	2738 (26%)	1260 (21%)	350 (30%)	232 (31%)	234 (40%)	193 (36%)	157 (32%)	146 (29%)	99 (30%)	67 (26%)
Underw	73 (1%)	35(1%)	13(1%)	2 (0%)	3 (1%)	5 (1%)	3 (1%)	8 (2%)	2 (1%)	2(1%)

*Note*: Class 0: No MM (No Multimorbidity); Class 1: HRT, STK (Heart Disease/Stroke); Class 2: AST, LUNG (Asthma/Lung Disease); Class 3: ARTH,HBP (Arthritis/Hypertension); Class 4: DEP, ARTH (Depression/Arthritis); Class 5: HBP,CATR,DIA (Hypertension/Cataracts/Diabetes); Class 6: PSY, DEP (Psychiatric Problems/Depression); Class 7: CAN (Cancer) and Class 8: ARTH, CATR (Arthritis/Cataracts).

### Linear Mixed Models

For both trajectories, the final model that best fitted the data was Model 3 which showed the lowest BIC, RMSEA < 0.01 and significant evidence from the log-likelihood ratio test where similar models were rejected. Means and standard deviations for immediate and delayed recall for each wave for the total sample and each class are given in Table 2.

**Table 2.** Means and Standard Deviations for Immediate and Delayed Recall for Maximal Sample Available at Each Wave

Immediate Recall	Wave 1 (*N* = 8711)	Wave 2 (*N* = 6428)	Wave 3 (*N* = 5426)	Wave 4 (*N* = 11104)	Wave 5 (*N* = 7409)	Wave 6 (*N* = 5992)	Wave 7 (*N* = 4688)
Total sample	5.43 (1.77)	5.62 (1.80)	5.64 (1.84)	5.64 (1.81)	5.63 (1.87)	5.66 (1.90)	5.63 (1.89)
Non-multimorbid	5.70 (1.70)	5.86 (1.73)	5.86 (1.80)	5.84 (1.76)	5.81 (1.82)	5.86 (1.84)	5.79 (1.84)
Class 1: Heart Disease/Stroke	4.88 (1.80)	5.04 (1.90)	5.09 (1.82)	5.07 (1.93)	5.18 (1.85)	5.14 (2.06)	5.07 (1.96)
Class 2: Asthma/Lung Disease	5.26 (1.73)	5.49 (1.78)	5.46 (1.85)	5.48 (1.83)	5.32 (1.94)	5.39 (1.96)	5.50 (2.01)
Class 3: Arthritis/Hypertension	5.25 (1.72)	5.37 (1.72)	5.44 (1.82)	5.37 (1.70)	5.35 (1.87)	5.21 (1.98)	5.30 (1.95)
Class 4: Depression/Arthritis	4.91 (1.83)	5.06 (1.89)	4.88 (1.90)	5.13 (1.82)	5.13 (1.91)	5.08 (1.95)	4.97 (1.93)
Class 5: Hypertension/Cataracts/Diabetes	4.88 (1.77)	5.10 (1.74)	5.10 (1.73)	5.13 (1.81)	5.10 (1.95)	5.02 (1.94)	5.03 (1.96)
Class 6: Psychiatric Problems/Depression	5.60 (1.73)	5.72 (1.90)	5.98 (1.73)	5.91 (1.82)	5.95 (1.75)	5.97 (1.68)	5.89 (1.80)
Class 7: Cancer	5.21 (1.63)	5.43 (1.85)	5.49 (1.77)	5.39 (1.78)	5.53 (1.90)	5.63 (1.81)	5.24 (1.97)
Class 8: Arthritis/Cataracts	4.81 (1.96)	5.22 (1.84)	4.97 (1.86)	4.98 (1.89)	5.05 (2.07)	4.82 (2.19)	5.17 (1.53)
Delayed Recall	Wave 1 (*N* = 11117)	Wave 2 (*N* = 7402)	Wave 3 (*N* = 5993)	Wave 4 (*N* = 4680)	Wave 5 (*N* = 8719)	Wave 6 (*N* = 6441)	Wave 7 (*N* = 5427)
Total sample	3.97 (2.10)	4.24 (2.11)	4.35 (2.17)	4.33 (2.14)	4.33 (2.20)	4.47 (2.15)	4.24 (2.21)
Non-multimorbid	4.30 (2.05)	4.55 (2.04)	4.62 (2.11)	4.58 (2.09)	4.60 (2.12)	4.70 (2.10)	4.44 (2.16)
Class 1: Heart Disease/Stroke	3.28 (2.15)	3.52 (2.21)	3.58 (2.30)	3.71 (2.25)	3.67 (2.24)	3.86 (2.20)	3.56 (2.25)
Class 2: Asthma/Lung Disease	3.79 (2.01)	4.14 (1.96)	4.17 (2.12)	4.19 (2.10)	3.92 (2.18)	4.16 (2.10)	4.03 (2.32)
Class 3: Arthritis/Hypertension	3.82 (2.00)	3.89 (1.99)	4.17 (2.05)	4.07 (2.12)	3.88 (2.19)	3.98 (2.15)	3.84 (2.23)
Class 4: Depression/Arthritis	3.29 (2.12)	3.47 (2.13)	3.50 (2.13)	3.49 (2.16)	3.64 (2.36)	3.73 (2.18)	3.38 (2.27)
Class 5: Hypertension/Cataracts/Diabetes	3.22 (2.08)	3.56 (2.09)	3.78 (2.06)	3.75 (2.05)	3.62 (2.13)	3.55 (2.22)	3.58 (2.33)
Class 6: Psychiatric Problems/Depression	4.18 (2.08)	4.45 (2.13)	4.61 (2.09)	4.63 (2.11)	4.62 (2.17)	4.82 (1.97)	4.60 (2.03)
Class 7: Cancer	3.69 (1.96)	4.04 (2.12)	4.26 (2.41)	3.99 (2.18)	4.02 (2.29)	4.50 (2.15)	3.88 (2.33)
Class 8: Arthritis/Cataracts	3.35 (2.10)	3.49 (2.06)	3.63 (2.23)	3.39 (2.07)	3.34 (2.35)	3.61 (2.26)	3.40 (2.16)

### Immediate Recall

For immediate recall, the unadjusted linear models (Model 1) showed that individuals recalled on average 5.79 words and decreased 0.05 points by year. At intercept level, we found that individuals with multimorbidity, regardless of their specific multimorbidity patterns, showed lower scores in immediate recall when compared with the group with no multimorbidity (except for Class 6: PSY, DEP). Pairwise post hoc analyses showed that each pattern was significantly different to the other patterns except for the following pairs (Class 2: AST, LUNG and Class 7: CAN; Class 3: ARTH, HBP and Class 1: HRT, STK; Class 3: ARTH, HBP and Class 5: HBP, CATR, DIA). When the model was adjusted by sociodemographics (Model 2), the association between multimorbidity and immediate recall was partially attenuated for most multimorbidity patterns and fully attenuated when additional adjustments were considered (Model 3), except for Class 4: DEP, ARTH which showed significantly lower scores for immediate recall.

At slope level, only individuals from Class 1: HRT, STK, Class 3: ARTH, HBP and Class 8: ARTH, CATR showed a faster decline over time compared with those individuals with no multimorbidity. Pairwise post hoc analyses showed that each of these patterns was significantly different to other patterns except for the following pairs (Class 1: HRT, STK and Class 4: DEP, ARTH; Class 1: HRT, STK and Class 2: AST, LUNG; Class 3: ARTH, HBP and Class 7: CAN). Once adjustments were included, the association between multimorbidity and change in immediate recall was fully attenuated (Table 3).

**Table 3.** Intercept and Slope Estimates for Multimorbidity Classes (reference category: no multimorbidity) for Immediate Recall Trajectories for Unadjusted and Adjusted Models

	Model 1: Unadjusted	Model 2: Adjusted by Sociodemographics	Model 3: Additionally Adjusted by BMI and Health Behaviors
Intercept			
Intercept	5.79*** (5.75, 5.831)	5.64*** (5.554, 5.72)	5.33*** (5.181, 5.48)
Multimorbidity patterns (ref: no_MM)			
Class 1: HRT, STK	−0.79 (−0.898, −0.677)***	−0.07 (−0.168, 0.03)	−0.03 (−0.125, 0.072)
Class 2: AST, LUNG	−0.43 (−0.559, −0.307)***	−0.12 (−0.223, −0.008)*	−0.08 (−0.191, 0.024)
Class 3: ARTH, HBP	−0.38 (−0.517, −0.248)***	0.03 (−0.092, 0.154)	0.04 (−0.081, 0.165)
Class 4: DEP, ARTH	−0.79 (−0.94, −0.632)***	−0.26 (−0.391, −0.126)***	−0.22 (−0.35, −0.084)**
Class 5: HBP, CATR, DIA	−0.75 (−0.899, −0.592)***	−0.15 (−0.291, −0.004)*	−0.13 (−0.277, 0.011)
Class 6: PSY, DEP	−0.11 (−0.265, 0.041)	−0.13 (−0.258, −0.004)*	−0.11 (−0.232, 0.021)
Class 7: CAN	−0.41 (−0.59, −0.235)***	−0.02 (−0.181, 0.131)	0.01 (−0.15, 0.161)
Class 8: ARTH,CATR	−0.67 (−0.891, −0.446)***	0.1 (−0.094, 0.293)	0.13 (−0.059, 0.325)
Slope			
Slope	−0.05 (−0.055, −0.036)***	−0.05 (−0.077, −0.031)***	−0.06 (−0.107, −0.016)**
Multimorbidity patterns (ref: no_MM)			
Class 1: HRT, STK	−0.07 (−0.102, −0.039)***	−0.01 (−0.043, 0.017)	−0.01 (−0.042, 0.018)
Class 2: AST, LUNG	−0.03 (−0.059, 0.004)	0.001 (−0.031, 0.031)	0.001 (−0.032, 0.03)
Class 3: ARTH,HBP	−0.04 (−0.08, −0.003)*	0.001 (−0.034, 0.039)	0.001 (−0.034, 0.04)
Class 4: DEP, ARTH	−0.02 (−0.057, 0.018)	0.001 (−0.036, 0.037)	0.001 (−0.035, 0.038)
Class 5: HBP, CATR, DIA	−0.04 (−0.086, 0.01)	0.02 (−0.029, 0.063)	0.02 (−0.028, 0.064)
Class 6: PSY, DEP	0.03 (−0.002, 0.066)	0.02 (−0.01, 0.056)	0.02 (−0.009, 0.056)
Class 7: CAN	−0.01 (−0.063, 0.036)	0.02 (−0.024, 0.068)	0.02 (−0.026, 0.067)
Class 8: ARTH, CATR	−0.1 (−0.159, −0.039)**	−0.04 (−0.095, 0.021)	−0.04 (−0.094, 0.022)

*Note*: *p < .05; **p < .01; ***p < .001.

### Delayed Recall

For delayed recall, the unadjusted linear model (Model 1) showed that individuals recalled on average 4.46 words and decreased 0.04 points by year. At intercept level, we found that individuals with multimorbidity, regardless of their specific multimorbidity patterns, showed lower scores in delayed recall when compared to the group with no multimorbidity (except for Class 6: PSY, DEP). Pairwise post hoc analyses showed that each pattern was significantly different to the other patterns except for the following pairs (Class 1: HRT, STK and Class 2: AST, LUNG; Class 2: AST, Lung with Class 5: HBP, CATR, DIAB or Class 8: ARTH, CATR; Class 3: ARTH, HBP with Class 7: CAN or Class 8: ARTH, CATR; Class 7: CAN and Class 8: ARTH, CATR). When the model was adjusted by sociodemographics, the association between multimorbidity and delayed recall was partially attenuated for most multimorbidity patterns and fully attenuated in the fully-adjusted model (Model 3). The only pattern that showed significantly lower scores for immediate recall at baseline after adjusting for sociodemographics, BMI and health behaviors was Class: 4: DEP, ARTH and Class: 5: HBP, CATR, DIA.

At slope level, individuals from Class 1: HRT, STK, Class 2: AST, LUNG; Class 3: ARTH, HBP, Class: 5: HBP, CATR, DIA and Class 8: ARTH, CATR showed a faster decline over time compared with those individuals with no multimorbidity. Pairwise post hoc analyses showed that each of these patterns was significantly different to other patterns except for the following pairs (Class 4: DEP, ARTH and Class 5: HBP, CATR, DIA; Class 1: HRT, STK with Class 2: AST, LUNG, Class 3: ARTH, HBP or Class 5: HBP, CATR, DIA). Once adjustments were included, the association between multimorbidity and change in delayed recall was fully attenuated (Table 4).

**Table 4.** Intercept and Slope Estimates for Multimorbidity Classes (reference category: no multimorbidity) for Delayed Recall Trajectories for Unadjusted and Adjusted Models

	Model 1: Unadjusted	Model 2: Adjusted by Sociodemographics	Model 3: Additionally Adjusted by BMI and Health Behaviors
Intercept			
Intercept	4.46*** (4.406, 4.507)	4.31*** (4.208, 4.408)	3.92*** (3.74, 4.097)
Multimorbidity patterns (ref: no_MM)			
Class 1: HRT, STK	−1.03 (−1.165, −0.893)***	−0.13 (−0.247, −0.005)*	−0.08 (−0.196, 0.045)
Class 2: AST, LUNG	−0.5 (−0.645, −0.354)***	−0.11 (−0.236, 0.02)	−0.07 (−0.198, 0.058)
Class 3: ARTH, HBP	−0.45 (−0.614, −0.283)***	0.07 (−0.068, 0.218)	0.09 (−0.055, 0.231)
Class 4: DEP, ARTH	−1.01 (−1.187, −0.831)***	−0.37 (−0.524, −0.216)***	−0.32 (−0.475, −0.168)***
Class 5: HBP, CATR, DIA	−0.99 (−1.176, −0.799)***	−0.22 (−0.389, −0.049)*	−0.2 (−0.372, −0.033)*
Class 6: PSY, DEP	−0.14 (−0.326, 0.051)	−0.18 (−0.34, −0.021)*	−0.15 (−0.311, 0.008)
Class 7: CAN	−0.56 (−0.791, −0.336)***	−0.07 (−0.268, 0.135)	−0.03 (−0.233, 0.169)
Class 8: ARTH, CATR	−0.9 (−1.161, −0.64)***	0.07 (−0.16, 0.297)	0.11 (−0.121, 0.337)
Slope			
Slope	−0.04 (−0.05, −0.028)***	−0.05 (−0.074, −0.023)***	−0.04 (−0.094, −0.004)*
Multimorbity patterns (ref: no_MM)			
Class 1: HRT, STK	−0.07 (−0.107, −0.038)***	−0.02 (−0.048, 0.018)	−0.02 (−0.049, 0.017)
Class 2: AST, LUNG	−0.04 (−0.073, −0.005)*	−0.01 (−0.044, 0.021)	−0.01 (−0.046, 0.019)
Class 3: ARTH, HBP	−0.06 (−0.096, −0.019)**	−0.01 (−0.048, 0.026)	−0.01 (−0.049, 0.025)
Class 4: DEP, ARTH	−0.01 (−0.049, 0.032)	0.01 (−0.033, 0.046)	0.01 (−0.033, 0.046)
Class 5: HBP, CATR, DIA	−0.06 (−0.113, −0.012)*	0.001 (−0.052, 0.046)	0.001 (−0.052, 0.046)
Class 6: PSY, DEP	0.03 (−0.009, 0.063)	0.02 (−0.018, 0.053)	0.02 (−0.019, 0.053)
Class 7: CAN	−0.01 (−0.073, 0.045)	0.03(−0.031, 0.081)	0.02 (−0.032, 0.079)
Class 8: ARTH, CATR	−0.11 (−0.177, −0.049)***	−0.05(−0.112, 0.012)	−0.05 (−0.112, 0.011)

*Note*: *p < .05; **p < .01; ***p < .001.

Similar results for immediate and delayed recall were found when sensitivity analyses were performed comparing complete-case and multiple-imputation, and when we assessed the survival effect by comparing results from samples with and without cohort members who died before Wave 7.

## Discussion

When we explored the different multimorbidity patterns in our sample, we found the following 8 classes from higher to lower prevalence: Class 1: Heart Disease/Stroke, Class 2: Asthma/Lung Disease, Class 3: Arthritis/Hypertension, Class 4: Depression/Arthritis, Class 5: Hypertension/Cataracts/Diabetes, Class 6: Psychiatric Problems/Depression, Class 7: Cancer and Class 8: Arthritis/Cataracts. Our findings share similarities with previous research that identified a clear cardiovascular disease pattern reflecting our Class 1 (4,26), a respiratory cluster reflected in our Class 2 (27) or a large group with hypertension and arthritis as characteristic features which are found in our Class 3 and known to be the most prevalent in the sister study of ELSA in the United States (28). Moreover, our methodological approach has allowed us to also identify other groups that overlap those found to be highly prevalent in previous research in the United Kingdom (1,2). Our classes were compared those individuals who had none or only one health condition (which we named no multimorbidity) and we found that, in general, individuals with multimorbidity were more likely to have lower SES which is consistent with previous research in multimorbidity in the United Kingdom (1,2). We also found that although there were sex differences across all classes, Class 4: Depression/Arthritis showed high prevalence of women which is common in clusters with physical and mental health comorbidities (2). This Class 4 was also found to be the class with lower levels of education and higher prevalence of obesity which suggest that this group might had higher probability of risk accumulation over their life course.

When we examined the association of the identified multimorbidity patterns with concurrent and subsequent immediate and delayed recall scores, we found that overall individuals with multimorbidity had lower scores in both tasks at baseline (except for the mental health group Class 6). Specifically, we found that at baseline all these groups showed lower scores in immediate recall when compared to the group with no multimorbidity but sociodemographic variables were considered (Model 2), the association between multimorbidity and immediate recall was partially attenuated for most multimorbidity patterns and fully attenuated when BMI and health behaviors were considered taken into account. These results are consistent with previous cross-sectional research (4–6,8). Moreover, these differences tend to be smaller when individuals have higher education levels, less social disadvantage background and engage in healthier behaviors such as moderate or vigorous physical activity. These results are in line with the cognitive reserve theory (29) and with life course epidemiology models (30) which sustain that engaging in healthy behaviors has a protective effect for cognitive performance in later adulthood. However, individuals in Class: 4: Depression/Arthritis still showed significantly lower scores for immediate recall than those individuals with no multimorbidity even after these adjustments. Further research should explore this group of people from a life-course perspective.

With regards to subsequent changes in the following 14 years, we found that although most of the population exhibits some decline over time in immediate and delayed recall tasks, the rate of this decline was only slightly accelerated for some classes when compared to the no multimorbidity group (Classes 1, 3, and 8 for immediate recall and Classes 1, 2, 3, 5, and 8 for delayed recall). However, once sociodemographic confounders were considered, these associations were fully attenuated. Although our study is not directly comparable, we find that most patterns with an accelerated decline included high blood pressure as one of the conditions of high prevalence, which is in line with the results found by Olaya et al. (31). Bratzke et al. (12) found no significant decline in memory for those assigned to the depression class (cholesterol, depression, and arthritis) or the cardiovascular class (cholesterol, hypertension, and arthritis). Our study found a significant decline for Class 3 (arthritis and hypertension) in unadjusted models and these differences might be associated differences in the assessment of memory, the methodological approach to capture change over time or the potential higher prevalence of cholesterol in their sample. On the other side, we found consistent results for those individuals in classes with depression. Previous research in the United States and European populations also found that depression stable patterns were not associated with faster decline in memory but patterns with depression onset over time seem to be associated with concurrent memory decline (15). Future studies considering changes in multimorbidity patterns over life might contribute to further our understanding of the complex association between depression and cognitive decline in individuals with multiple chronic health conditions. On the other hand, Wei et al. (10) found that in U.S. population each point increase in a multimorbidity index that they developed was associated with faster decline in immediate recall tasks but not delayed recall tasks. Again, we cannot compare our results as their index has a greater number of health conditions and these differ, in addition interpretability is limited to quantity. Our results differed from those reported by Melis et al. (7) who found a faster decline in cognitive performance over 5 years but it should be noted that their sample consisted only of individuals with dementia while our study focused on dementia free population and aimed to draw conclusions from normative UK population.

One of the main strengths of the present study is that it goes beyond understanding multimorbidity in terms of quantity as most of previous research (5–7,9), which it has interesting public health implications. Our findings show that individuals over 50 years old which can be classified as our Class 4: Depression/Arthritis and Class 5: Hypertension/Cataracts/Diabetes which represent 12% and 10% of our UK representative sample are likely to have lower levels of memory compared to individuals with other patterns of multimorbidity and individuals with no multimorbidity. Potential underlying shared mechanisms such as vascular pathways for individuals in Class 5 or the impact of antidepressants or pain medication for individuals in Class 4 should be addressed in future studies. With regards to the potential use of these classes to identify individuals at higher risk of an accelerated decline (which in turn can be a predictor of future cognitive impairment), our findings highlight that within this age range, any differences are driven by sociodemographics confounders such as age and education. Future research should investigate further patterns of multimorbidity and their association with memory earlier in life.

Some limitations should be acknowledged. First, this study has only focused in memory as a proxy of cognitive performance and different results could be found when other cognitive domains are considered. For example, Fabri et al. (32) found a faster decline in executive function, processing speed, and verbal fluency but not in visual or verbal memory. Future studies with longer follow-ups in other cognitive domains should be performed. Comparisons with trajectories in executive function would be very interesting given that previous research has found that changes in executive function precede changes in memory in aging populations (33). Second, although a wide range of conditions were considered these were self-reported and restricted to those collected in ELSA and different patterns might arise if we replicated using primary health records. This study has also limited data on treatments, medication, or management of these conditions, which might also have an impact in our outcome. For example, individuals who have their blood pressure very well controlled and optimally managed could have a slower decline than those that were not optimally managed. Future research investigating the association between multimorbidity and cognitive decline should explore the role of treatments, medication intake or management. In addition, covariates were only considered at baseline but changes over time in these should also be considered in future research. Fourth, drinking could be also a relevant health behavior to consider in future studies. Unfortunately, drinking was not included in the study as a large majority (over 90%) had drunk alcohol and the self-reported frequency of alcohol consumption was highly variable and subject to reporting bias (34). Fifth, when interpreting results of longitudinal studies one should consider that the sample might be only representative of healthy survivors in the population (35). We performed sensitivity analyses in order to take this potential bias into account; however, the multimorbidity patterns identified might be representative of a healthy survivor sample at each age group.

To sum up, we found that individuals of a representative UK sample can be classified in 8 different classes according to their patterns of multimorbidity. Individuals in these classes differ significantly from individuals with no multimorbidity and between them in terms of sociodemographics, BMI and health behaviors. When we examined whether individuals classified on those patterns differ in their memory scores cross-sectionally or in their rate of decline, we found that individuals in Class 4: Depression/Arthritis and Class 5: Hypertension/Cataracts/Diabetes have lower scores in recall tasks at baseline but not in their rate of decline. Future studies should further investigate potential shared mechanisms in individuals in Class 4 and Class 5 and potential drivers of decline earlier in life. In addition, we found that most multimorbidity patterns including hypertension showed an accelerated decline which was fully attenuated when potential confounders were included. Overall, these findings suggest that individuals with multiple chronic health conditions—and specifically those with certain combinations—are more likely to have lower levels of memory compared to individuals with none or one single chronic health condition and their memory scores tend to differ between combinations. However, when we examined their association with subsequent decline, it seems that sociodemographics or health behaviors have a key role to understand who is more likely to be at risk of an accelerated decline.

## Supplementary Material

glab009_suppl_Supplementary_Materials

glab009_suppl_Supplementary_Table_S1

## Data Availability

Data were extracted from the English Longitudinal Study of Aging which is public available dataset. More details in https://www.elsa-project.ac.uk/.
