# Supplementary figures and images for: Multimorbidity Patterns and Memory Trajectories in Older Adults: Evidence From the English Longitudinal Study of Aging
Source: J Gerontol A Biol Sci Med Sci. 2021 Jan 15;76(5):867–75. doi: 10.1093/gerona/glab009 (PMC8087269; doi:10.1093/gerona/glab009)

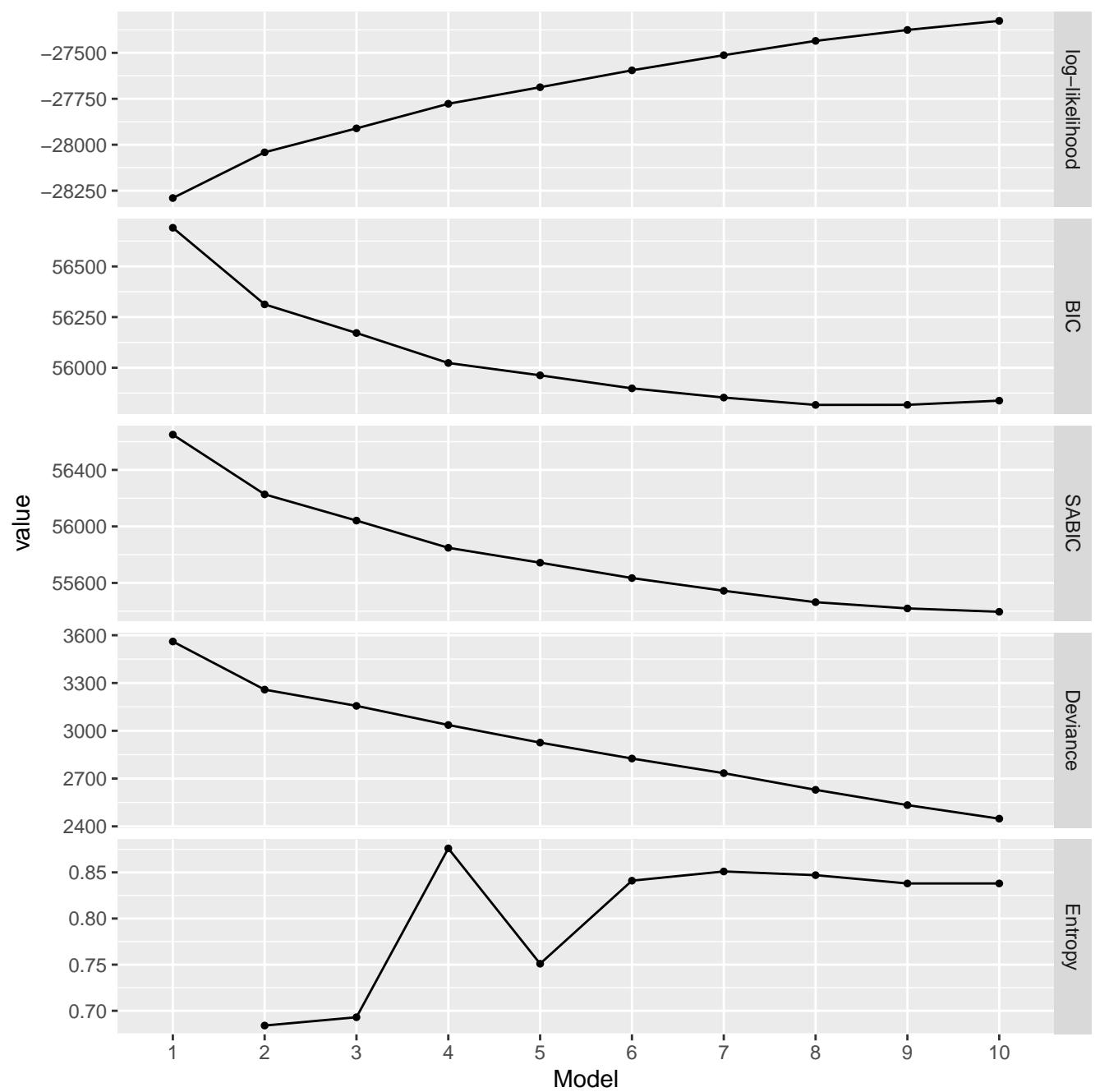

Supplement: glab009_suppl_Supplementary_Materials [file glab009_suppl_supplementary_materials.pdf]
