# Supplementary material for: Multimorbidity Patterns and Memory Trajectories in Older Adults: Evidence From the English Longitudinal Study of Aging
Source: J Gerontol A Biol Sci Med Sci. 2021 Jan 15;76(5):867–75. doi: 10.1093/gerona/glab009 (PMC8087269; doi:10.1093/gerona/glab009)
Supplement: glab009_suppl_Supplementary_Table_S1 [file glab009_suppl_supplementary_table_s1.docx]

*Supplemental Table 1.* Multinomial regression of each MM cluster against the reference non-multimorbid clusters

|  |  | Class:1:  HRT,STK | Class 2:  AST, LUNG | Class: 3:  ARTH,HBP | Class 4:  DEP,ARTH | Class: 5: HBP,  CATR,DIA | Class 6:  PSY,DEP | Class 7:  CAN | Class 8:  ARTH,CATR |
| --- | --- | --- | --- | --- | --- | --- | --- | --- | --- |
| **Age** |  | 5.252^***^ | 1.388^***^ | 3.746^***^ | 1.313^***^ | 4.809^***^ | -2.159^***^ | 4.074^***^ | 5.980^***^ |
|  |  | (4.718,5.787) | (0.796,1.979) | (3.051,4.440) | (0.634,1.992) | (4.058,5.561) | (-2.931,-1.386) | (3.187,4.960) | (4.929,7.031) |
| **Gender** (ref.=female) |  |  |  |  |  |  |  |  |  |
| Male |  | 0.146^*^ | -0.389^***^ | -0.419^***^ | -0.472^***^ | 0.373^***^ | -0.472^***^ | -0.535^***^ | -0.158 |
|  |  | (-0.006,0.299) | (-0.563,-0.214) | (-0.622,-0.216) | (-0.680,-0.265) | (0.156,0.589) | (-0.685,-0.259) | (-0.795,-0.275) | (-0.452,0.136) |
| **Smoking status**  (ref.=never smoked) | |  |  |  |  |  |  |  |  |
| Ever-smoked |  | 0.347^***^ | 0.291^***^ | -0.001 | 0.370^***^ | 0.269^**^ | 0.301^***^ | 0.478^***^ | 0.204 |
|  |  | (0.188,0.506) | (0.114,0.467) | (-0.196,0.194) | (0.161,0.579) | (0.045,0.493) | (0.084,0.519) | (0.214,0.742) | (-0.089,0.497) |
| **Physical Activity** (ref.=None) |  |  |  |  |  |  |  |  |  |
| Light |  | -0.139 | -0.028 | 0.432^*^ | -0.074 | 0.04 | -0.038 | -0.203 | 0.019 |
|  |  | (-0.426,0.147) | (-0.376,0.320) | (-0.034,0.899) | (-0.442,0.294) | (-0.415,0.496) | (-0.497,0.421) | (-0.692,0.287) | (-0.496,0.535) |
| Moderate |  | -0.866^***^ | -0.714^***^ | 0.083 | -0.746^***^ | -0.24 | -0.593^***^ | -0.830^***^ | -0.509^**^ |
|  |  | (-1.112,-0.620) | (-1.015,-0.412) | (-0.331,0.497) | (-1.067,-0.424) | (-0.625,0.144) | (-0.985,-0.200) | (-1.250,-0.411) | (-0.961,-0.057) |
| Vigorous |  | -1.254^***^ | -1.005^***^ | -0.235 | -1.271^***^ | -0.659^***^ | -0.934^***^ | -0.995^***^ | -1.003^***^ |
|  |  | (-1.521,-0.987) | (-1.325,-0.685) | (-0.669,0.199) | (-1.629,-0.912) | (-1.070,-0.247) | (-1.348,-0.521) | (-1.441,-0.549) |  |
| **Marital status** (ref.=partnered) | |  |  |  |  |  |  |  |  |
| Single |  | -0.052 | 0.14 | -0.073 | 0.515^***^ | -0.057 | 0.453^***^ | 0.018 | -0.003 |
|  |  | (-0.217,0.113) | (-0.048,0.327) | (-0.292,0.147) | (0.306,0.725) | (-0.294,0.180) | (0.228,0.679) | (-0.258,0.295) | (-0.309,0.304) |
| **Wealth** (ref.= highest quintile) | |  |  |  |  |  |  |  |  |
| Quintile 1 |  | 0.636^***^ | 0.681^***^ | 0.327^*^ | 0.968^***^ | 0.293 | 1.064^***^ | 0.15 | 0.618^**^ |
|  |  | (0.379,0.894) | (0.387,0.976) | (-0.016,0.669) | (0.584,1.352) | (-0.066,0.652) | (0.714,1.414) | (-0.295,0.596) | (0.122,1.114) |
| Quintile 2 |  | 0.628^***^ | 0.498^***^ | 0.441^***^ | 0.839^***^ | 0.343^**^ | 0.233 | 0.297 | 0.515^**^ |
|  |  | (0.384,0.873) | (0.214,0.782) | (0.128,0.755) | (0.461,1.216) | (0.008,0.678) | (-0.133,0.599) | (-0.114,0.708) | (0.030,1.000) |
| Quintile 3 |  | 0.329^***^ | 0.338^**^ | 0.292^*^ | 0.649^***^ | 0.195 | 0.316^*^ | 0.278 | 0.464^*^ |
|  |  | (0.080,0.578) | (0.055,0.620) | (-0.018,0.603) | (0.266,1.032) | (-0.138,0.528) | (-0.029,0.661) | (-0.120,0.677) | (-0.015,0.944) |
| Quintile 4 |  | 0.101 | 0.218 | 0.209 | 0.478^**^ | 0.036 | 0.129 | 0.437^**^ | 0.231 |
|  |  | (-0.152,0.355) | (-0.064,0.500) | (-0.100,0.518) | (0.085,0.871) | (-0.299,0.371) | (-0.219,0.477) | (0.065,0.809) | (-0.258,0.721) |
| **Education** (ref.=less than secondary) | |  |  |  |  |  |  |  |  |
| Tertiary |  | -0.178 | 0.062 | -0.271 | -0.720^***^ | -0.249 | 0.522^***^ | 0.414^**^ | 0.061 |
|  |  | (-0.468,0.112) | (-0.233,0.356) | (-0.660,0.117) | (-1.177,-0.263) | (-0.646,0.147) | (0.184,0.861) | (0.009,0.818) | (-0.457,0.578) |
| Upper_secondary |  | 0.021 | -0.152 | 0.109 | -0.374^***^ | -0.006 | 0.183 | 0.062 | -0.202 |
|  |  | (-0.142,0.184) | (-0.342,0.039) | (-0.102,0.319) | (-0.598,-0.151) | (-0.234,0.222) | (-0.053,0.419) | (-0.216,0.340) | (-0.524,0.120) |
| **BMI** (ref.=Normal) |  |  |  |  |  |  |  |  |  |
| Obese |  | 0.510^***^ | 0.409^***^ | 0.908^***^ | 0.549^***^ | 0.616^***^ | 0.402^***^ | 0.346^**^ | 0.236 |
|  |  | (0.351,0.668) | (0.230,0.589) | (0.713,1.103) | (0.346,0.752) | (0.397,0.835) | (0.182,0.623) | (0.077,0.616) | (-0.077,0.549) |
| Underweight |  | -0.044 | -1.05 | -0.142 | 0.123 | -0.37 | 0.46 | -0.993 | -0.145 |
|  |  | (-0.847,0.760) | (-2.502,0.402) | (-1.387,1.103) | (-0.850,1.097) | (-1.824,1.083) | (-0.525,1.444) | (-3.003,1.017) | (-1.609,1.319) |
